# Tissue integration in the CAM assay, collagen intensity and near-interface cell density data for novel emulsion electrospun DegraPol®-Bakuchiol fiber meshes that were characterized by FTIR and for fiber thickness and pore size

**DOI:** 10.1016/j.dib.2026.113130

**Published:** 2026-08-03

**Authors:** Julia Rieber, Gabor Kadler, Tiziano A. Schweizer, Hendrik Koliwer-Brandl, Gabriella Meier Bürgisser, Pietro Giovanoli, Johanna Buschmann

**Affiliations:** a Division of Plastic Surgery and Hand Surgery, University Hospital of Zurich, 8091, Zurich, Switzerland; b Department of Cranio-Maxillo-Facial and Oral Surgery, University Hospital Zurich, University of Zurich, 8091, Zurich, Switzerland; c Institute of Medical Microbiology, University of Zurich, 8006, Zurich, Switzerland

**Keywords:** Chicken embryo, Electrospinning, Bakuchiol, Polymer, Collagen expression

## Abstract

To develop bioactive electrospun membranes capable of controlled Bakuchiol release, we engineered an emulsion-electrospun DegraPol® (DP) polymer mesh. The structural integrity of the scaffolds was characterized through detailed fiber morphology and porosity analysis. FTIR spectra were taken. To evaluate the biological response in vivo, the Bakuchiol functionalized meshes were integrated into the chorioallantoic membrane (CAM) assay of the avian embryo. Tissue interaction was assessed via Masson-Goldner trichrome staining, providing semi-quantitative data on tissue integration and collagen deposition intensity. Furthermore, total cell density within the immediate peri‑implant microenvironment was quantified using Haematoxylin-Eosin (H&E) histological sections. In addition, the bacterial adhesion on the scaffold was tested with a *Staphylococcus aureus*

strain. All experimental groups were evaluated against pure DP fiber meshes as a control to isolate the specific effects of Bakuchiol.

## Graphical abstract

**Figure:**
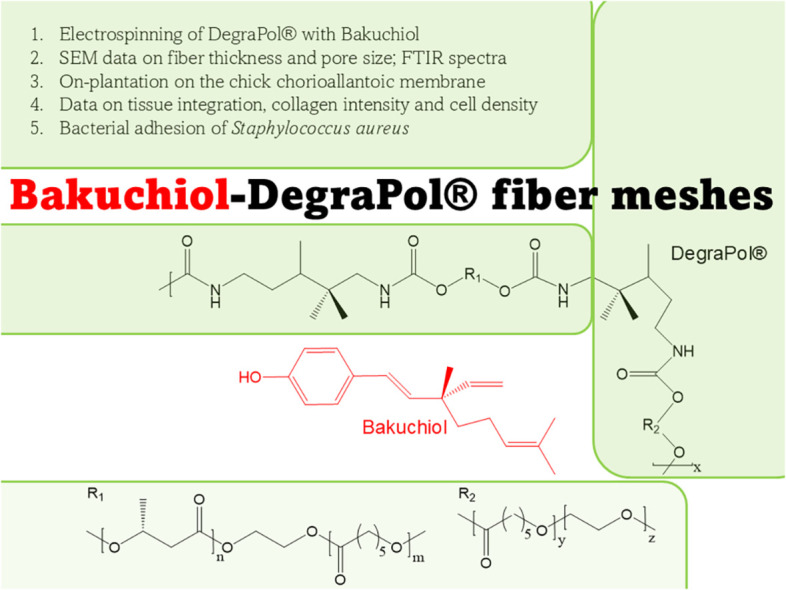

Specifications Table

**Table:** 

Subject	Material science, electrospinning, cell-material interactions, in vivo response towards novel material
Specific subject area	A novel mesh had been fabricated by emulsion electrospinning to incorporate Bakuchiol into DegraPol® (DP) polymer fibres. Via scanning electron microscopy (SEM), the fiber thickness and pore size was assessed. FTIR spectra were taken. Then, the DP-Bakuchiol meshes were planted onto the CAM assay. After one week, tissue integration, collagen intensity and cell density directly adjacent to the implant were determined. Finally, bacterial adhesion to the scaffolds was tested.
Data format	The data consist of raw and analysed data.
Type of data	Excel files of the type *.xlsx* files.
Data collection	Data collection was performed by analysis of scanning electron micrographs (SEM) of the DP-Bakuchiol fiber meshes. Histological analysis was performed in H&E-stained sections (total cell density – quantitative analysis) and in Masson Goldner Trichrome stained sections (tissue integration and collagen intensity, both semi-quantitative analyses, providing contingency tables with frequency of occurrence).
Data source location	All data were collected in Zurich, Switzerland, at the University Hospital Zurich in the laboratories of the Department for Plastic Surgery and Hand Surgery. The SEM images were taken at the University of Zurich Irchel, Switzerland. The bacterial adhesion experiments were performed at the Institute of Medical Microbiology, University of Zurich.
Data accessibility	Repository name: Mendeley Data Data identification number: doi:10.17632/sjps5fypy9.1 Direct URL to data: Data on cell density, tissue integration and collagen intensity of fibrous electrospun DegraPol-Bakuchiol scaffolds in the CAM assay, bacterial adhesion of Staphylococcus aureus; with data on FTIR, fiber thickness and porosity of the scaffolds - Mendeley Data
Related research article	Electrospun DegraPol® Meshes with Incorporated Bakuchiol: Characterization and Tissue Integration In Ovo—A Pilot Study

## 1 Value of the Data

• Our fiber thickness and porosity data can be reused, in case other polymers than DegraPol® are used to fabricate bioactive Bakuchiol containing meshes.
• The fiber thickness and porosity data can be used for comparison, if the same components are used for electrospinning, however, if parameters like distance to the collector, flow rate of polymer-Bakuchiol solution etc. are varied and changed.
• The tissue integration data can be used when other phenolic compounds than Bakuchiol are incorporated into emulsion electrospun meshes; our data represent reference points for other phenolic antibacterial antioxidant moieties incorporated in electrospun meshes.
• The total cell density at the interface of the material and the chorioallantoic membrane can be re-used to compare other materials to the DegraPol®-Bakuchiol system.
• The bacterial adhesion of *Staphylococcus aureus* to the scaffolds can be used for comparison with other bacterial strains.

## 2 Background

Bakuchiol belongs to the category of phenolic phytochemicals that can be easily incorporated in polymer fibers via emulsion electrospinning [1]. Given Bakuchiol’s antibacterial, antiviral and anti-inflammatory actions, such electrospun Bakuchiol containing and releasing fiber meshes may be used for wound healing approaches [2]. Specifically, we provide data on a formulation combining the co-polymer DegraPol® with Bakuchiol via ethanol-in-oil emulsion electrospinning, where we assessed the fiber thickness and the pore size. Furthermore, data on the material-cell interactions are covered by chorioallantoic membrane (CAM) assay data, such as tissue integration, collagen intensity in the tissue that grew into the fiber mesh, and total cell density at the interface between the mesh and the chorioallantoic membrane. Finally, we also provide data on the susceptibility of the electrospun material to contamination by bacteria, specifically for *Staphylococcus aureus*.

## 3 Data Description

The data are stored as five Microsoft Excel files (Microsoft Corporation, Redmond, WA, USA) (.xlsx files) in a Mendeley Data repository service entitled Data on cell density, tissue integration and collagen intensity of fibrous electrospun DegraPol-Bakuchiol scaffolds in the CAM assay, bacterial adhesion of Staphylococcus aureus; with data on FTIR, fiber thickness and porosity of the scaffolds - Mendeley Data.

Also, images of the CAM assay before and after excision of the scaffolds are provided.

The five excel files are named as follows: (i) Porosity_Fiberthickness_Bakuchiol.xlsx; (ii) Cell density at interface.xlsx; (iii) Contingency tables_Integration and Collagen intensity.xlsx; (iv) Bakuchiol_FTIR.xlsx; and (v) DegraPol_Bakuchiol_Staphylococcus aureus.xlsx respectively.

### 3.1 Fiber thickness and pore size data

**File** Porosity_Fiberthickness_Bakuchiol.xlsx

This excel file contains 4 sheets, called *Fibers_Bakuchiol, Pores_Bakuchiol, Fibers_DP*, and *Pores_DP*. The four sheets are equally structured. Columns A and K define the surface (either as inner or outer sufrace); columns B and L give the filename; columns C and M indicate the replicate number is; columns D and N represent the fiber thickness or the pore size, both given in [μm]. Columns E and O present the calculated mean; while F and P show the calculated the standard deviation of the mean; columns G and Q demonstrate the calculated mean per conditions, while for H and R, the standard deviation of the mean per condition is presented.

### 3.2 CAM assay data: total cell density

**File** Cell density at interface.xlsx

**Fig. 1:**
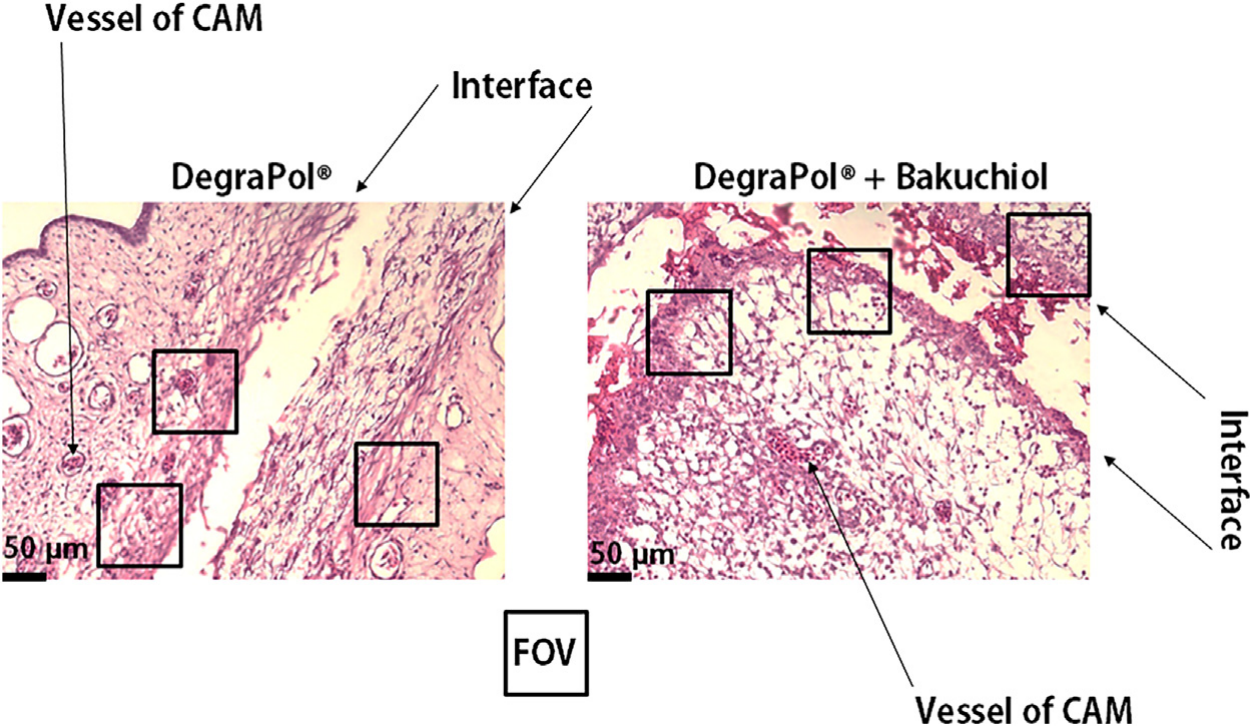
Two H&E-stained histological sections; on the left side for a pure DegraPol® fiber mesh, on the right side for a DegraPol®+Bakuchiol fiber mesh on the CAM after 1 week. Fields of view (FOVs) are indicated as black squares and are located at the interface between the scaffold and the CAM. Moreover, exemplary vessels are indicated by arrows. Finally, the interfaces are also indicated by arrows.

This excel file contains 1 sheets, called *Total cell density at interface*. In this sheet, column A gives the title; column B exhibits instructions on abbreviations and the size of the fields of view (FOVs), in which the cells were counted – as total cell numbers (Fig. 1). Column C then indicates the number of a specific FOV (always three FOVs were examined); columns d-G present the corresponding cells counted in each FOV for the pure DegraPol® fiber mesh (control). Then, in column I, the sample numbers are shown in light blue; columns J and give the calculated mean and the calculated standard deviation of the mean. For columns L to S, the same is shown as for columns D to K, but this time for the DegraPol®+Bakuchiol fiber mesh, the bioactive fiber mesh.

### 3.3 Tissue integration and collagen intensity data

**File** Contingency tables_Integration and Collagen intensity.xlsx

This excel file contains one sheet, called *Tabelle 1* and provides two contingency tables for the collagen intensity as assessed semi-quantitatively (upper table) and for the tissue integration, also assessed semi-quantitatively (lower table). Column A specifies the kind of material (with DP referring to DegraPol®), column B shows the frequency for Level 0, while in columns C to F, the frequency of occurrence for Levels 0.5, 1.0, 1.5, and 2.0, are presented. Column G shows the sum of all frequencies per line.

### 3.4 FTIR spectra

**File** Bakuchiol_FTIR.xlsx

This excel sheet contains three sheets, called *20240920_DP1_161023; 20240920_DP_Bakuchiol_100624*, and *C—O Ratio*, respectively. While in the first two sheets column A represents the wavenumber (cm^–1^) and column B the corresponding absorbance at this specific wavenumber; in the third sheet, column A gives the experimental group name, column B the name of the replicate, column C the absorbance for the C=O double bond, column D the absorbance of the C—O single bond and column E the calculated ratio of the absorbances of the C—O-to-C=O ratio.

### 3.5 Bacterial adhesion

**File** DegraPol_Bakuchiol_Staphylococcus aureus.xlsx

This excel file contains one sheet entitled *Bacterial adhesion experiments*. Column A shows the date of the two experiments (day, month, year). Columns B and C represent the number of inoculated bacterial CFUs at the beginning of the experiment for DegraPol® (DP) and DegraPol® plus Bakuchiol (DP + BK), respectively. Columns D and E show the CFU counts of adhered bacteria to the DP meshes after 30 min of incubation with the inoculum. Columns F and G show the relative adhesion of bacteria after 30 min of incubation for the DP meshes.

## 4 Experimental Design, Materials and Methods

### 5.1 Electrospinning and SEM analysis for fiber thickness and pore size

The DP + Bakuchiol fiber meshes were electrospun with an ethanol-in-oil emulsion, according to a protocol reported earlier [1]. The fiber thickness and the pore size were measured in SEM images, where diagonal lines were drawn – and the fibres crossing these lines were included [3]. Also pore sizes were measured on the basis of these lines.

### 4.2 CAM assay experiments: total cell density at the interface

After onplantation of the DP or the DP + Bakuchiol samples for 1 week in the CAM assay as reported earlier for other types of scaffolds [4], the H&E stained sections were analyzed for the cell density at the interface between CAM and scaffold – with 3 FOVs per sample (Fig. 1).

### 4.3 Tissue integration and collagen intensity data

After staining the histological sections with Masson Goldner Trichrome, we semi-quantitatively assessed the tissue integration (five levels from 0.0, 0.5, 1.0, 1.5, and 2.0 for low until high tissue integration) and the collagen intensity within the scaffold where tissue grew into the scaffold (also five levels from 0.0, 0.5, 1.0, 1.5, and 2.0 for low until high collagen intensity) [1].

### 4.4 Fourier transformed infrared spectroscopy (FTIR)

FTIR spectra of pure and Bakuchiol-loaded DP tubes were acquired using a Varian 640 spectrometer with a Golden Gate diamond ATR, ranging from 600 to 4000 cm^–1^, with a resolution of 4 cm^–1^, and 64 scans. Data were normalized to the C=O peak at 1720 cm^–1^ and the intensity ratio of the C—O to C=O peaks were calculated.

### 4.5 Bacterial adhesion

**Fig. 2:**
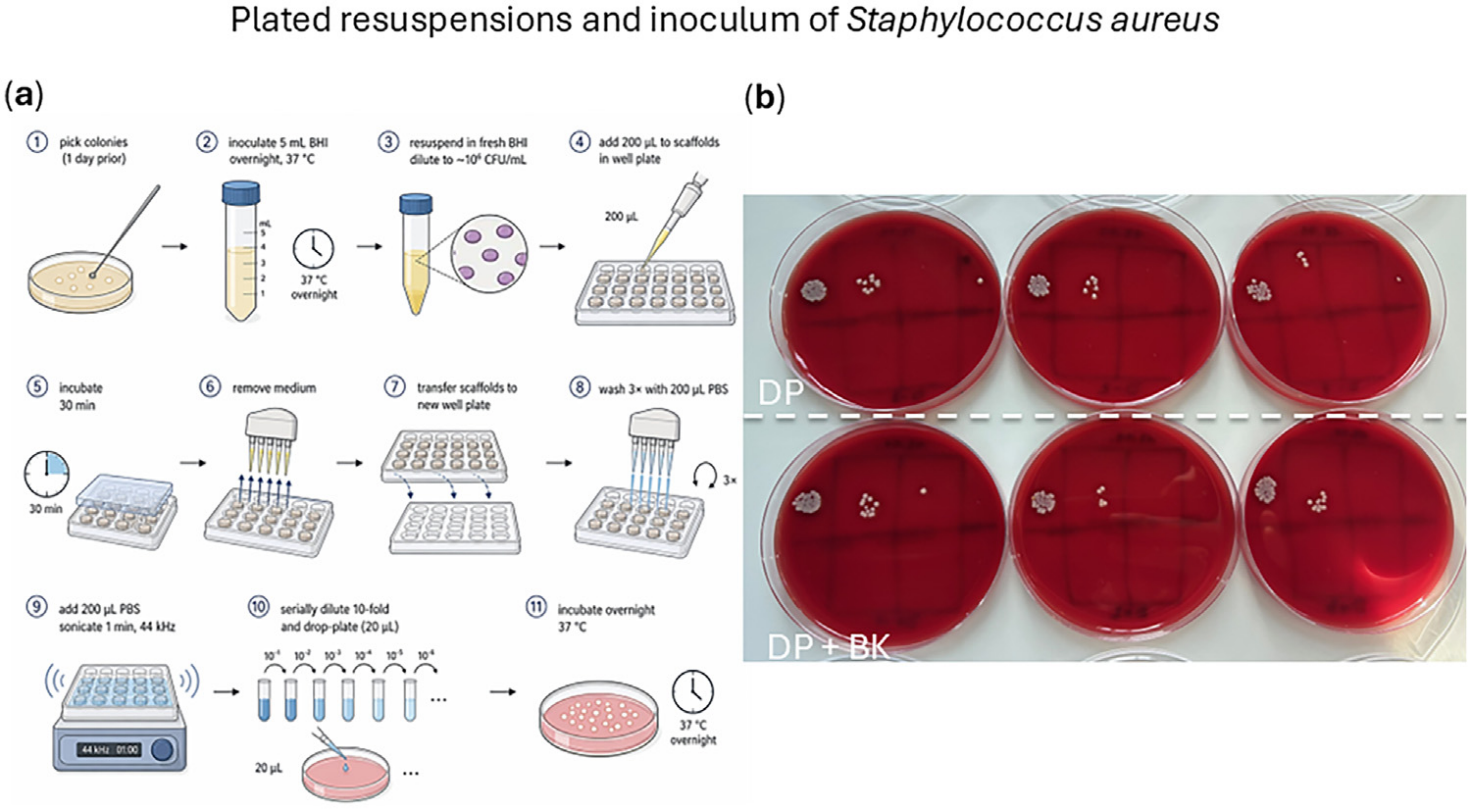
(**a**) Schematic depiction of the workflow. (**b**) Representative images of CFUs enumeration on agar plates from inoculated DegraPol® (DP, top row) and DP + 0.3 μg Bakuchiol per disc (DP + BK, bottom row) after sonication. Each quadrant represents a different dilution. Shown are three technical replicates from one biological replicate. Panel A was created using FigPad “Text to Figure” (GPT Image 2, accessed on 02.07.2026).

The selected bacterial strain *S. aureus* ATCC 29,213 (Fig. 2) was grown on Columbia sheep blood agar plates (bioMérieux, Mary-L’Etoile, France) at 37 °C without CO_2_. One day prior to the adhesion assay, multiple colonies were picked from the agar plate, inoculated in 5 mL of brain heart infusion medium Bacto™ brain heart infusion (BHI) bouillon (Becton Dickinson, Bacto™, New York, USA) and incubated overnight at 37 °C. On the day of the bacterial adhesion assay, the bacterial culture was resuspended in fresh BHI and subsequently diluted to approximately 10^6^ colony forming units per mL (CFUs/mL). Aliquots of 200 μL of the bacterial suspension were added to the scaffolds placed in 96-well plates and incubated for 30 min. At the desired time point, the medium was removed from the wells, the scaffolds transferred to a new 96-well plate and washed 3 × with 200 μL phosphate-buffered saline (PBS). Next, 200 μL of PBS was added to the scaffolds which were sonicated for 1 min at 44 kHz. After sonication, the bacterial suspensions were serially diluted 10-fold and drop-plated (20 µl) on agar plates. The plates were incubated overnight at 37 °C, after which CFUs/mL were determined. Two biological replicates were carried out in technical triplicates.

## Limitations

The 7-day timeframe of the CAM assay precluded the assessment of long-term tissue integration and collagen intensity. Additionally, the absence of a longitudinal time course restricted our insights into the dynamics of the process.

## Ethics Statement

The study was conducted in accordance with the veterinary office of the Canton Zurich, Switzerland, with the ethical approval under licence No. 255/15.

## CRediT Author Statement

**Julia Rieber:** Conceptualization, Methodology, Data curation, Investigation, Writing – Original draft preparation, Writing – Reviewing and Editing. **Gabor Kadler**: Investigation, Methodology, Data curation, Writing – Reviewing and Editing. **Tiziano Schweizer**: Conceptualization, Writing – Reviewing and Editing. **Hendrik Koliwer-Brandl**: Methodology, Resources, Writing – Reviewing and Editing. **Gabriella Meier Bürgisser**: Data curation, Data analysis, Writing – Reviewing and Editing. **Pietro Giovanoli**: Supervision, Writing – Reviewing and Editing. **Johanna Buschmann**: Conceptualization, Supervision, Writing – Original draft preparation, Writing – Reviewing and Editing, Fund raising, Administration.

## Data Availability

Mendeley DataData on cell density, tissue integration and collagen intensity of fibrous electrospun DegraPol-Bakuchiol scaffolds in the CAM assay, bacterial adhesion of Staphylococcus aureus; with data on FTIR, fi (Original data).
